# A systematic review and meta-analysis on the efficacy and safety of finerenone in the progression of heart failure

**DOI:** 10.3389/fphar.2025.1575307

**Published:** 2025-09-12

**Authors:** Shengtian Peng, Peipei Li, Zhixi Yu, Beibei Du, Ping Yang

**Affiliations:** China-Japan Union Hospital, Jilin University, Changchun, China

**Keywords:** finerenone, heart failure, efficacy, safety, meta-analysis

## Abstract

**Aims:**

Finerenone, a kind of mineralocorticoid receptor antagonist (MRA), may benefit heart failure (HF) patients as MRAs are established effective therapies for HF. Many studies have confirmed the drug’s effectiveness in treating kidney disease. However, the efficacy and safety of finerenone on HF remain unclear. Therefore, this systematic review and meta-analysis was conducted to assess the preliminary efficacy and safety of finerenone in HF treatment.

**Methods:**

This systematic review and meta-analysis included randomized controlled trials (RCTs) involving adults with heart failure, diabetes, or chronic kidney disease (CKD) treated with finerenone. The major outcomes were the risk of HF occurrence or worsening and hospitalization due to HF, whereas the secondary outcomes included cardiovascular death and all-cause mortality. Data were extracted and analyzed following PRISMA guidelines, and risk of bias was evaluated using the Cochrane Handbook. This review was registered with the International Prospective Register of Systematic Reviews (PROSPERO; CRD42024612580).

**Results:**

Six RCTs (n = 21, 295) were included. Finerenone was associated with a lower risk of HF occurrence or worsening and hospitalization due to HF than placebo [risk rate (RR): 0.81; 95% confidence interval (CI): 0.76–0.87; P < 0.00001]. However, no prominent differences were found in cardiovascular death (RR: 0.93; 95% CI: 0.83–1.03; P = 0.18) or all-cause mortality (RR: 0.94; 95% CI: 0.87–1.02; P = 0.11). Safety analysis indicated a reduced risk of serious adverse reactions (RR: 0.93; 95% CI: 0.90–0.98; P = 0.005) and discontinuation of the study medication due to adverse events (RR: 1.14; 95% CI: 1.01–1.30; P = 0.04).

**Conclusion:**

Finerenone appears to decrease the risk related to HF occurrence and progression, particularly in patients with CKD and diabetes, but its impact on overall mortality remains uncertain. The potential benefits need to be balanced against the risk of adverse effects. Further research is essential to explore optimal dosing and treatment duration.

## 1 Introduction

Heart failure (HF), characterized by compromised cardiac output on account of impaired ventricular systolic function, is a worldwide health issue with significant morbidity and mortality rates. The progression of heart failure is impacted by various reasons, including some diseases (Zoccali et al., 2023; Sano, 2020; Triposkiadis et al., 2022; Beghini et al., 2024) such as diabetes, chronic kidney disease (CKD), and other cardiovascular conditions. Despite advances in medical therapies, the management of heart failure remains challenging, and there is a continuous need for novel and effective treatments.

At present, the main treatment methods for heart failure include “quadruple therapy” (Swedberg et al., 2010; Yancy et al., 2013). Finerenone, a selective mineralocorticoid receptor antagonist (MRA), has presented new promise in therapeutic management of HF. This drug was used for the treatment of kidney diseases because of its pharmacological characteristics, such as CKD and diabetes (Beghini et al., 2024), and numerous animal studies and clinical trials have demonstrated its efficacy (Yao et al., 2023; Kolkhof et al., 2014; Zhu Z. et al., 2023; González-Blázquez et al., 2018). As kidney disease and heart disease are closely related (Zoccali et al., 2023; Sano, 2020), this drug, as an aldosterone receptor antagonist, can antagonize the mineralocorticoid receptor (MR) to reduce fibrosis of the heart and kidney caused by MR overactivation (Kolkhof et al., 2014; Agarwal et al., 2021; Grune et al., 2018). However, robust evidence on the efficacy and safety of finerenone in therapeutic management of HF remains limited. Therefore, we systematically searched and screened relevant clinical randomized controlled trials (RCTs), extracted the number of any events related with HF or the risk factor of HF such as CKD and diabetes, and then conducted a systematic review and meta-analysis on the efficacy and safety of finerenone in the occurrence and development of heart failure, aiming to provide references for clinical practice.

## 2 Methods

**FIGURE 1:**
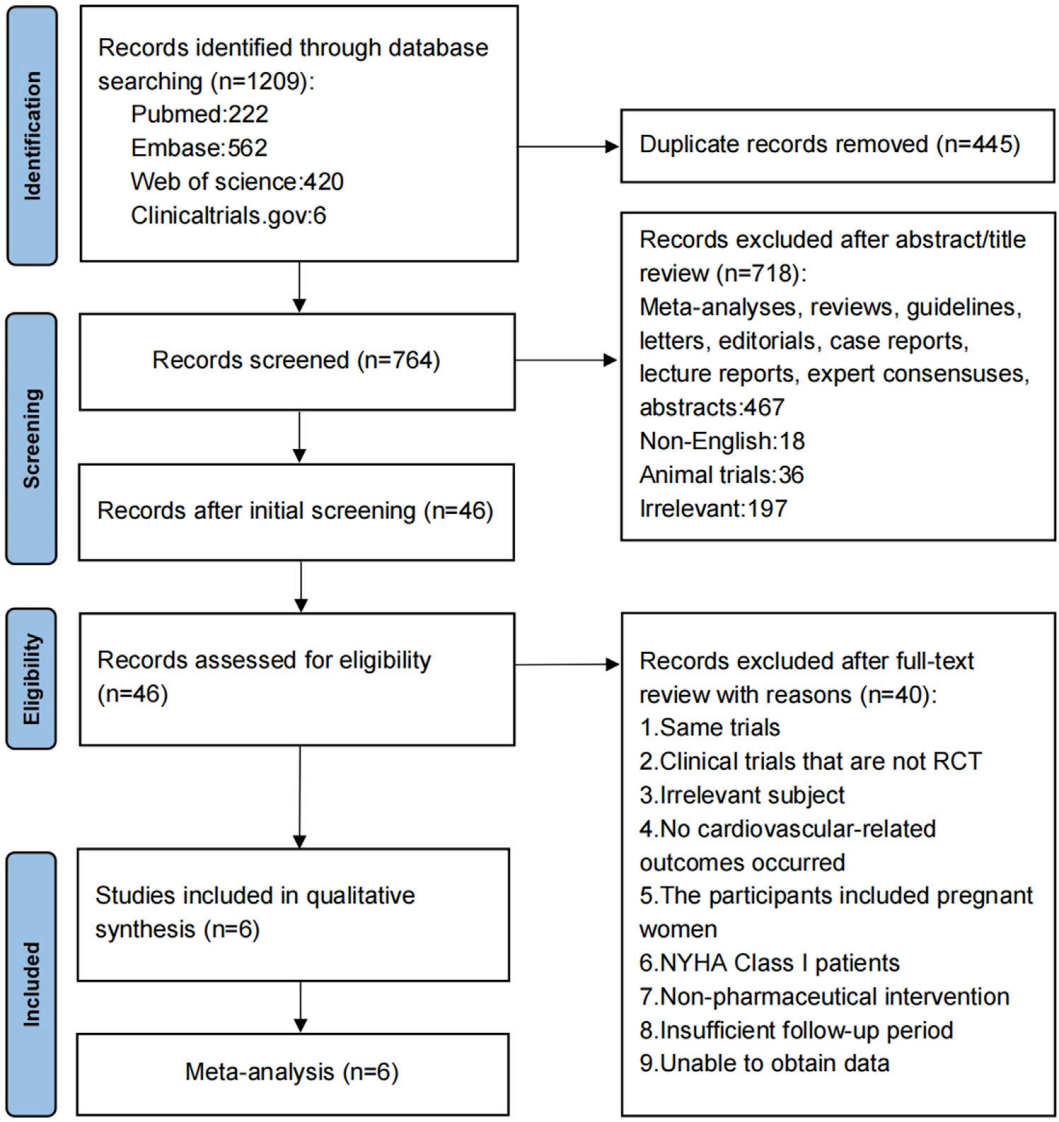
PRISMA systematic review flow diagram.

Data sources and search strategies, data acquisition, inclusion and exclusion criteria, outcome measures, assessment of quality, and statistical methods in this research were performed according to the Guidelines for Systematic Review and Meta-Analysis (PRISMA), and the study flow was designed according to PRISMA flow diagram standards (Figure 1). This research was registered with the International Prospective Register of Systematic Reviews (PROSPERO: CRD42024612580).

### 2.1 The inclusion and exclusion criteria

Inclusion and exclusion criteria were based on PICOTS principles (Table 1).

**TABLE 1:** Inclusion and exclusion criteria.

	Inclusion criteria	Exclusion criteria
Participant (P)	1. Adults aged 18 years or older 2. With NYHA class II–IV or diabetes or CKD	Pregnant women
Intervention (I)	Finerenone	Non-pharmaceutical intervention
Control (C)	Placebo/matched dose of standard treatment	
Outcome (O)	Efficacy: 1. HF occurrence or worsening or hospitalization due to HF, 2. cardiovascular death, and 3. all-cause mortality Safety: 1. Serious adverse reactions and 2. discontinuation of the study medication or trial regimen due to adverse events	
Time (T)	Treating time ≥4 weeks	
Study (S)	RCTs (the resulting data were made public, and trials were registered on clinical.gov)	Clinical trials that are non-randomized controlled trials

The inclusion criteria were as follows: (1) adults who were with NYHA class II–IV, diabetes, or CKD (diagnostic criteria: eGFR <60 mL/min/1.73 m^2^ and duration at least 3 months) and aged 18 years or older; (2) intervention group treated with finerenone; (3) reporting major outcomes: heart failure occurs or worsens, or hospitalization due to HF, cardiovascular death (the cardiovascular death, as referred to here, includes deaths caused by myocardial infarction, coronary artery diseases, and myocardial ischemia), and all-cause mortality; (4) treating time ≥4 weeks; (5) RCTs (the resulting data were made public, and trials were registered on clinical.gov).

Exclusion criteria were as follows: 1) pregnant women; 2) non-pharmaceutical intervention; 3) non-randomized controlled trials, animal studies, reviews, meta-analyses, case reports, reviews, abstracts of meetings, letters, guidelines, expert consensuses, and non-English studies.

### 2.2 The strategy of search and the sources of data

We searched the Web of Science, PubMed, and Embase using keywords related with finerenone and heart failure to identify concerned articles published from the time of database establishment to 10 Nov 2024. We also manually added articles from ClinicalTrials.gov and the latest research. The index term of ClinicalTrials.gov was “finerenone”, and the filters were set as “with results”. The index terms of PubMed, Embase, and Web of Science include “finerenone”, “BAY 94-8862”, “kerendia”, “Heart Failure”, “Cardiac Failure”, “Heart Decompensation”, “Decompensation, Heart”, “Congestive Heart Failure”, “Myocardial Failure”, “Left Sided Heart Failure”, and “Right Sided Heart Failure”.

### 2.3 Selection of studies, data extraction, and quality assessment

Two independent research workers were responsible for the section of study, based on titles, abstracts, and full texts. According to a unified data extraction table, two reviewers extracted the essential information and data from each eligible trial. Any disagreements were resolved through consultation or discussion with a third party.

Risk of bias was assessed following the domains suggested in the Cochrane Handbook for Systematic Review of Interventions, version 6.5 (Higgins JPT, Green S, eds. Cochrane Handbook for Systematic Reviews of Interventions [version 6.5, 2024] http://handbook.Cochrane.org/. Accessed 20 Nov 2024), including selection bias, performance bias, detection bias, attrition bias, reporting bias, and other bias.

### 2.4 Data processing and statistical analysis

Regarding efficacy, the outcomes were the risk related to the occurrence or worsening of HF, hospitalization due to HF, and cardiovascular death and all-cause mortality. In terms of safety, the outcomes were serious adverse reactions and the discontinuation of the study medication or trial regimen due to adverse events. Combined risk rate (RR) values and 95% confidence interval (CI) were used as effect indicators, and *P-value* < 0.05 indicated statistical significance. Q tests and I^2^ statistics were used to evaluate heterogeneity. If *P-value* for Q test >0.1 and I^2^ < 50%, there was no statistically significant heterogeneity, and the Mantel–Haenszel and fixed-effect models were used for combined analysis. We used a funnel plot and Begg’s test to assess the potential publication bias. All analyses followed the intention-to-treat principle and were conducted using RevMan 5.4.1 (The Cochrane Collaboration).

## 3 Results

### 3.1 Screening

A total of 1,209 relevant papers, 5 trials, and 1 latest research were obtained initially. Six clinical randomized controlled trials (n = 21,295) were finally included through screening. The screening process and outcomes are displayed in Figure 1.

### 3.2 Clinical characteristics of included studies and evaluation of bias risk

**FIGURE 2:**
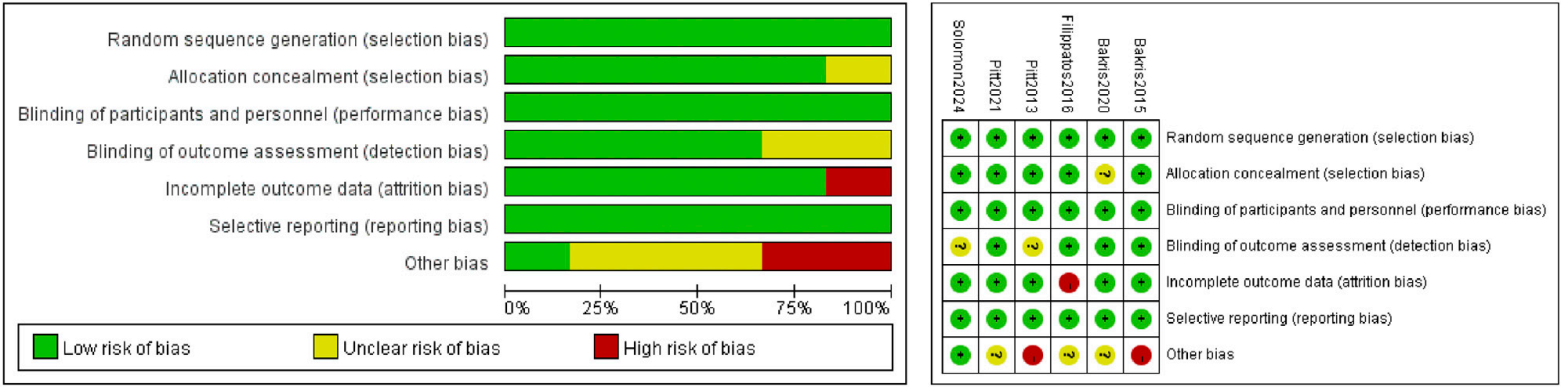
Quality assessment of included RCTs.

This meta-analysis included six (Ba et al., 2015; Filippatos et al., 2016; Pitt et al., 2013; Pitt et al., 2021; Bakris et al., 2020; Solomon et al., 2024) RCTs, including a total of 21,295 participants. A total of 11,347 patients were treated with finerenone and 9, 948 were treated with placebo. The clinical characteristics of each research are displayed in Table 2. We used the Cochrane bias risk assessment tool and found that the total risk of bias was low. The results are shown in Figure 2.

**TABLE 2:** Baseline characteristics of included RCTs.

Author (year)	NCT number	Population	Duration of treatment and follow-up	Sample size (n)		Male (%)		Age [mean ± SD /mean (range)]		Intervention		Outcome
				Experimental	Control	Experimental	Control	Experimental	Control	Experimental	Control	
Bakris et al. (2020)	NCT01874431	Diabetes	90 days	727	94	78.4	73.4	64.2 ± 9.2	63.3 ± 8.7	1.25–20 mg finerenone	Placebo	2, 3, 4, and 5
Filippatos et al. (2016)	NCT01807221	HF and diabetes	120 days	834	221	77.3	76.9	70.8 ± 10.2	72.4 ± 9.9	2.5–20 mg finerenone	Eplerenone	1, 2, 3, 4, and 5
Pitt et al. (2013)	NCT01345656	HF and CKD	4 weeks	264	128	79.6	72.1(40-89)		2.5–10 mg finerenone		Spironolactone/placebo	1
Pitt et al. (2021)	NCT02545049	Diabetes	3.4 years	3686	3666	68.6	70.3	64.1 ± 9.7	64.1 ± 10.0	10–20 mg finerenone	Placebo	1, 2, 3, 4, and 5
Bakris et al. (2020)	NCT02540993	Diabetes and CKD	2.6 years	2833	2841	68.9	71.5	65.4 ± 8.9	65.7 ± 9.2	10–20 mg finerenone	Placebo	1, 2, 3, 4, and 5
Solomon et al. (2024)	NCT04435626	HF	32 months	3003	2998	54.9	54.1	71.9 ± 9.6	72.0 ± 9.7	20–40 mg finerenone	Placebo	1, 2, 3, and 4

1. Heart failure development, worsening or hospitalization due to heart failure. 2. Cardiovascular death. 3. All-cause mortality. 4. Serious adverse reaction. 5. Discontinuation of the study medication or trial regimen due to adverse events.

### 3.3 Meta-analysis

This research is focused on the efficacy and safety of finerenone in the population with risk factors (diabetes and CKD) for developing HF or those with HF. Efficacy indicators include heart failure occurrence or worsening, hospitalization due to heart failure (result 1), cardiovascular death (result 2), and all-cause mortality (result 3). Safety indicators include serious adverse reactions (result 4) and discontinuation of the study medication or trial regimen due to adverse events (result 5).

#### 3.3.1 Efficacy analysis

**FIGURE 3:**
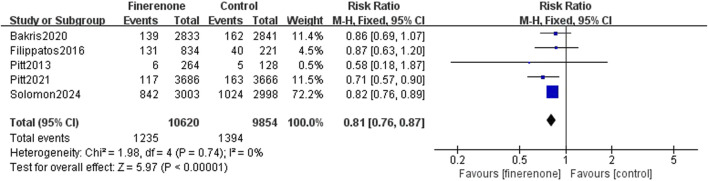
Incidence of HF occurrence, worsening or hospitalization due to HF.

**FIGURE 4:**
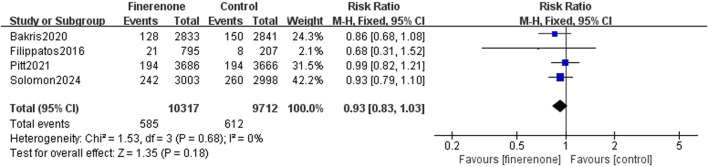
Occurrence of cardiovascular death events.

**FIGURE 5:**
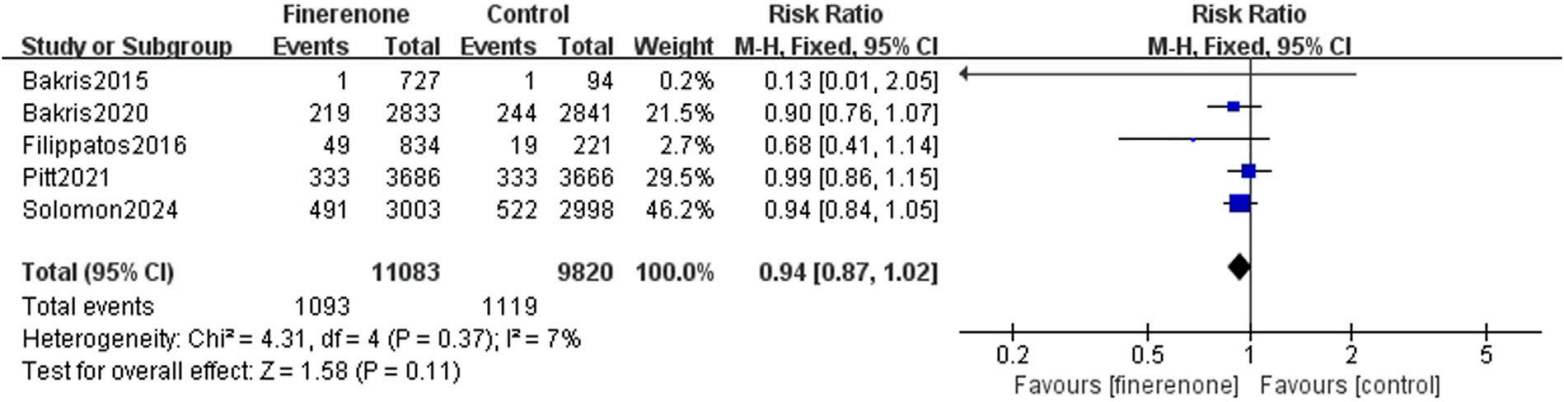
All-cause mortality.

Results 1–3: five RCTs reported events, which included heart failure development or worsening and hospitalization due to heart failure, and meta-analysis revealed that patients using finerenone had a lower risk than those using placebo (RR: 0.81; 95% CI: 0.76–0.87; I^2^ = 0%; P < 0.00001) (Figure 3). Five RCTs reported events about cardiovascular death, and the analysis revealed no statistically significant variations between the groups on the risk related to cardiovascular death (RR: 0.93; 95% CI: 0.83–1.03; I^2^ = 0%; P = 0.18) (Figure 4). A total of five studies described all-cause death events, and statistical analysis demonstrated no significant intergroup discrepancies in the incidence of all-cause death events in the finerenone group compared with the placebo (RR: 0.94; 95% CI: 0.87–1.02; I^2^ = 7%; P = 0.11) (Figure 5).

#### 3.3.2 Safety analysis

**FIGURE 6:**
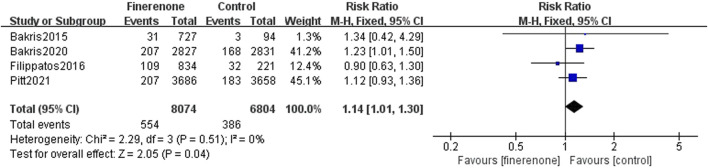
Serious adverse reactions.

**FIGURE 7:**
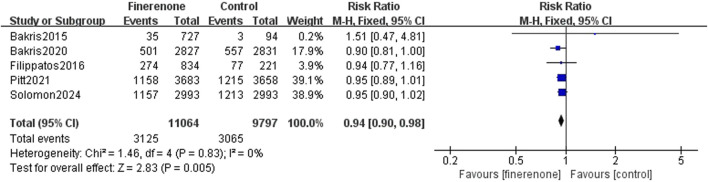
Discontinuation of the study medication or trial regimen due to adverse events.

Five studies reported serious adverse reactions, and it is obvious that there are fewer adverse reactions when using finerenone (RR: 0.93; 95% CI: 0.90–0.98; I^2^ = 0%; P = 0.005) (Figure 6). Four trials reported discontinuation of the study medication or trial regimen due to adverse events, and meta-analysis results showed that side effects were more likely in those using finerenone than in those in the placebo group (RR: 1.14; 95% CI: 1.01–1.30; I^2^ = 0%; P = 0.04) (Figure 7).

#### 3.3.3 Sensitivity analysis and publication bias

For sensitivity analysis, we conducted a one-by-one elimination method (every research was excluded separately). We found that the results of meta-analyses of studies 1, 2, and 4 were not affected by a single study. However, result 3 showed a statistically significant difference after excluding NCT002540993 (RR: 0.95; 95% CI: 0.87–1.04; I^2^ = 26%; P = 0.25), NCT02545049 (RR: 0.92; 95% CI: 0.83–1.00; I^2^ = 13%; P = 0.06) or NCT04435626 (RR: 0.94; 95% CI: 0.84–1.05; I^2^ = 30%; P = 0.26).

**FIGURE 8:**
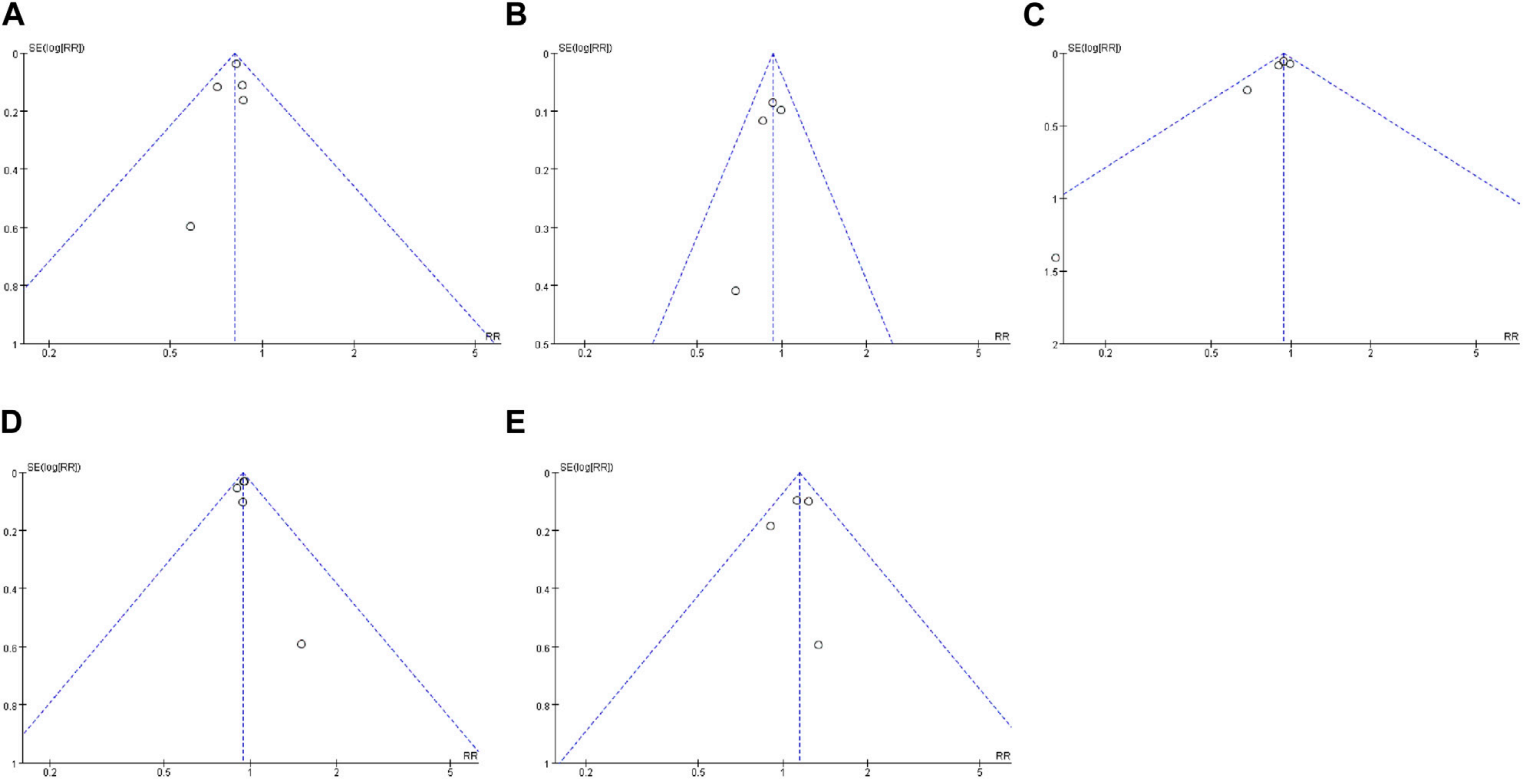
Funnel plot. **(A)** Incidence of HF; **(B)** death events; **(C)** mortality; **(D)** adverse reaction; **(E)** discontinuation.

It is difficult to explain whether these studies have publication bias from the funnel plot (Figure 8). Therefore, we used STATA 14.0 software to conduct Begg’s test to assess potential publication bias. The results of outcome indicators 1–5 were as follows: P = 0.807, 0.734, 0.086, 1.000, and 1.000. The findings indicate a low probability of publication bias in these studies.

## 4 Discussion

Finerenone has certain advantages over traditional corticosteroid receptor blockers, such as lower occurrence of hyperkalemia than spironolactone (Ba et al., 2015; Filippatos et al., 2016; Pitt et al., 2013; Rico-Mesa et al., 2020), high affinity for MR, and more promising as a corticosteroid receptor blocker (Amazit et al., 2015). Compared with previous similar studies (Zhu Y. et al., 2023; Yang et al., 2023), this research excluded the studies that did not use the blind method and included the latest research results (Solomon et al., 2024) with a larger sample size. The results were divided into two categories of efficacy and safety, and the feasibility of finerenone for therapeutic management of heart failure was evaluated. More detailed and specific results are provided on relevant indicators for the occurrence and development of heart failure.

In response to the results of the efficacy (Figures 4–6), we considered the following possible reasons: 1) in NCT002540993, the diagnosis of CVD was retrospective diagnosis, not formally assessed at the baseline. Meanwhile, this research focused on the population with later-period CKD. 2) Differences in race play a role in the results. 3) In NCT02545049, some patients were treated with other drugs, such as SGLT2 inhibitors and GLP-1 receptor agonists.

A serious adverse reaction is any untoward medical occurrence at any dose that results in death or is life-threatening, according to the ICH E2A guidelines (International Council for Harmonisation of Technical Requirements for Pharmaceuticals for Human Use, 2024). It is interesting that there is a lower risk of serious adverse reactions in the group using finerenone. This may be because the patients selected for the randomized controlled trials included in this study had a risk of deteriorating cardiac function. The greatest challenge these patients face, posing life-threatening risks or even death, lies in their cardiovascular system rather than in the side effects caused by the drug itself. In addition, according to the outcomes of the efficacy, finerenone can reduce the risk of developing, worsening, or being hospitalized due to HF. Therefore, result 4 and result 5 are not contradictory, and it is possible that the protective effect comes from the drug’s ability to protect the heart and kidney functions (Kolkhof et al., 2014; Agarwal et al., 2021; Grune et al., 2018; Lv et al., 2023), rather than from its low incidence of side effects. Although finerenone reduced SAR risk, the higher discontinuation rate may reflect non-serious but intolerable adverse effects (e.g., hyperkalemia). Heterogeneity in SAR definitions across trials could also contribute to this discrepancy. Therefore, it is valuable to explore more regarding serious adverse reactions caused by finerenone itself.

This research also has some limitations. Some studies excluded people with heart failure and selected people with kidney diseases such as CKD and diabetes. Moreover, because the number of included studies was relatively small, differences in sample sizes between studies can also have an impact. Consequently, the generalizability of the results may be influenced by the size of the sample and methodological quality of the included studies. Additionally, a subgroup analysis based on the dosage was not conducted in this study. The relation between the dosage and the effect of finerenone remains unclear. Future studies should stratify patients by finerenone dose (e.g., 10 mg vs. 20 mg or low, middle, and high dose) to assess efficacy and safety. In addition, the heterogeneity of study design and patient populations may limit the ability to analyze certain subgroups.

In summary, we analyzed existing studies with more than 4-week follow-up time. The results suggest that finerenone can reduce the risks related to the occurrence and development of HF. However, the appropriate dose and duration of finerenone treatment for HF remain unknown and need to be verified in future studies.

## 5 Conclusion

Our meta-analysis demonstrates that finerenone significantly reduces the composite risk of HF development, worsening, or hospitalization compared to placebo (RR: 0.81; 95% CI: 0.76–0.87; P < 0.00001), aligning with its proposed cardioprotective role. However, no statistically significant reduction was observed for cardiovascular death (RR: 0.93; 95% CI: 0.83–1.03; P = 0.18) or all-cause mortality (RR: 0.94; 95% CI: 0.87–1.02; P = 0.11), suggesting that its primary benefit may lie specifically in mitigating HF events. Sensitivity analysis indicated that the overall mortality results were not robust to the exclusion of specific trials, potentially influenced by study design, population characteristics (e.g., race and CKD stage) or concomitant medications. Regarding safety, finerenone was associated with a lower risk of serious adverse reactions (RR: 0.93; 95% CI: 0.90–0.98; P = 0.005) but a higher risk of discontinuation due to adverse events (RR: 1.14; 95% CI: 1.01–1.30; P = 0.04). Thus, its efficacy in HF must be balanced against potential adverse effects.

## Data Availability

The original contributions presented in the study are included in the article/supplementary material. Further inquiries can be directed to the corresponding author.
